# Effects of different types of Tai Chi intervention on motor function in older adults: a systematic review

**DOI:** 10.1007/s40520-024-02894-5

**Published:** 2025-01-22

**Authors:** Xu Fan, Kim Geok Soh, Chan Yoke Mun, Kim Lam Soh

**Affiliations:** 1 Department of Sport Studies, Faculty of Education Studies, Universiti Putra Malaysia, Selangor, 43400 Malaysia; 2 Department of Dietetics, Faculty of Medicine and Health Sciences, Universiti Putra Malaysia, Selangor, 43400 Malaysia; 3 Department of Nursing, Universiti Putra Malaysia, Serdang, Selangor Malaysia

**Keywords:** Tai Chi, Motor function, Older adults, Balance, Gait

## Abstract

**Background:**

Tai Chi (TC) is widely acknowledged for its positive impact on improving motor function in older adults. Nevertheless, limited research has directly compared the effects of different TC styles on older adults with functional impairments.

**Objective:**

This study aimed to assess the impact of different TC styles on motor function in older adults with functional impairments.

**Method:**

We searched five databases—PubMed, Scopus, Chinese National Knowledge Infrastructure (CNKI), Web of Science, and Wiley Online Library—including studies published up to September 2024. The selection of literature adhered to PRISMA guidelines, with quality assessment independently carried out by two researchers.

**Results:**

Fourteen studies met the inclusion criteria for this review. The analysis revealed that TC interventions for functionally impaired older adults primarily employed Yang-style, Sun-style, Chen-style, and simplified-style TC. The populations studied included individuals with mild cognitive impairment (MCI), nonspecific low back pain (NS-LBP), preclinical disabilities, chronic diseases, poor balance, osteoarthritis (OA), Parkinson’s disease (PD), sarcopenia, and those at risk of falls. The findings indicated that motor function in functionally impaired older adults were closely linked to balance, gait, mobility, strength, and fall rates. Among the various TC styles, Yang-style was the most frequently utilised intervention.

**Conclusion:**

This review examined four types of TC interventions and found strong evidence supporting the effectiveness of Yang-style TC in improving motor function in older adults with functional impairments. Additionally, five assessment methods—Single-Leg Stance (SL), Six-Minute Walk Test (6MWT), Timed Up and Go Test (TUGT), Chair Stand Test (CST), and Fall Efficacy Scale (FES)—were identified as suitable for evaluating this population. Based on the findings, it is recommended that individuals with functional impairments engage in Yang-style 24-movement TC, with an intervention duration of 12 weeks, practicing two to five times a week for 60 min each session.

**Supplementary Information:**

The online version contains supplementary material available at 10.1007/s40520-024-02894-5.

## Introduction

As the global population ages, there is growing concern about enhancing the quality of life (QoL) for older adults, particularly those with functional impairments [1, 2]. Motor function, which enables the body to move and maintain posture, depends on the coordinated work of muscles and the nervous system [3]. The natural decline in biological systems such as reduced muscle strength and balance has a direct impact on motor function, which is essential for maintaining independence and overall well-being in older adults [4–6]. Motor dysfunction, however, is a common challenge among older adults, often seen as decreased mobility, a heightened risk of falls, and a reduced capacity to perform daily activities. These issues can further impact mental health and QoL [7, 8].

Tai Chi originated in China and is a branch of Chinese martial arts [9, 10]. Over time, it has evolved into various styles such as Chen, Yang, Wu/Hao, Wu, and Sun [11, 12], with Chen style being the oldest [12, 13]. TC is a moderate-intensity mind-body exercise that numerous studies have shown to improve physical performance in older adults, including balance, gait, strength, and mobility [14–17]. However, inconsistencies in findings have been reported across studies, attributed in part to variations in TC styles and forms used [18]. For instance, one study suggested that an 8-form Yang-style TC intervention did not improve gait abilities in Parkinson’s disease (PD) patients [19], whereas another study found positive effects from a 24-form Yang-style intervention [20, 21]. Differences in TC forms and intervention populations have led to inconsistencies in intervention results [22], making it challenging to identify the most suitable TC style for older adults, particularly those with varying functional impairments.

Numerous studies have examined the effects of TC on motor function in older adults, yet it remains unclear which TC style best supports those with functional impairments, such as MCI, PD, OA, NS-LBP, fall risk, preclinical disabilities, chronic diseases, and sarcopenia. Each of these conditions presents unique functional challenges and impacts key physical health indicators, including balance, gait, mobility, strength, and risk of falls. For instance, musculoskeletal conditions often lead to reduced balance and mobility, while cognitive and neurological impairments can significantly affect gait and overall functional performance [23, 24]. This review systematically investigates the effects of different TC interventions on these physical health indicators in older adults with functional impairments. By focusing on specific outcomes such as balance, gait, mobility, strength, and fall prevention, this study seeks to identify the most suitable TC interventions to enhance motor function in this population.

## Materials and methods

### Protocol and registration

This systematic review was registered in PROSPERO with registration number CRD 42,024,562,526. It was conducted in strict adherence to the Preferred Reporting Items for Systematic Reviews (PRISMA) guidelines [25].

### Eligibility criteria

Table 1 presents the inclusion criteria established according to the PICOS principles (Population, Intervention, Comparison, Outcome, and Study Design):

Table 1 outlines the inclusion and exclusion criteria. The criteria for this systematic review were established based on the PICOS (Population, Intervention, Comparison, Outcomes, and Study Design) principles. (1) Participants included older adults aged 60 years or older, particularly those with functional impairments related to motor function, such as MCI, PD, OA, NS-LBP, sarcopenia, disabilities affecting movement, and those at high risk of falls. (2) The intervention group must have undergone TC training, incorporating various styles (e.g., Yang, Chen, Sun, simplified), aimed at improving motor function, such as balance, gait, strength, mobility, and fall risk. (3) The control group could have received any alternative intervention (e.g., stretching exercises or usual care) or no intervention. (4) Only randomised controlled trials (RCTs) reporting outcomes related to motor function were included. (5) Studies published in either Chinese or English were considered.

Exclusion criteria included: (1) studies involving healthy older adults. (2) not focusing on exercise outcomes. (3) non-RCT designs (e.g., observational studies, case reports, or theoretical studies). (4) studies published in languages other than Chinese or English. (5) Tai Chi styles and forms (Neither of them mentioned).

**Table 1:** Inclusion and exclusion criteria

	Items	Detail
**Inclusion criteria**	Participants	No restriction on gender; participants must be aged ≥ 60 years with functional impairments, including MCI, PD, OA, NS-LBP, sarcopenia, disabilities, or at high risk of falls.
	Intervention	Tai Chi intervention (including Yang, Chen, Sun, and Simplified styles)
	Comparison	Control group, no exercise or exercise group
	Outcome	Balance, gait, mobility, strength, falls
	Study designs	RCT
**Exclusion Criteria**	Participants	Healthy older adults
	Intervention	Tai Chi intervention period of less than 6 weeks
	Outcome	Studies that do not primarily focus on outcomes related to motor function, such as those that concentrate solely on mental health
	Study designs	Non-RCT designs (observational studies, case reports, or cohort studies)

### Literature search

This systematic review conducted searches across five databases: PubMed, Scopus, CNKI, Web of Science, and Wiley Online Library. The literature search encompassed articles from inception to September 2024. PubMed (MeSH) terms and the following keywords were used in the search: “Tai Ji” OR “Tai-ji” OR “Tai Chi” OR “Chi Tai” OR “Tai Ji Quan” OR “Ji Quan Tai” OR “Quan Tai Ji” OR “Taiji” OR “Taijiquan” OR “T’ai Chi” OR “Tai Chi Chuan” AND “motor” “movement” OR “motion” OR “mobility” OR “function” OR “performance” AND “old people” OR “elderly” OR “senior*” OR “old adult*” OR “aged” OR “older people” OR “older adults” OR “geriatric.” Some keywords are derived from published reviews. Please refer to the supplementary materials for the specific search strategy of each database.

### Study selection

All included literature was imported into EndNote reference management software to remove duplicate records. Subsequently, two researchers independently assessed each article’s titles, abstracts, and keywords to determine eligibility based on the inclusion criteria. For articles that passed this initial screening, full-text assessments were performed. In cases of discrepancies between the two researchers, a third researcher was consulted to achieve consensus.

### Data extraction and quality assessment

Data extraction included: (1) author and publication year; (2) sample size, gender, and age; (3) health status; (4) intervention type, method, frequency, duration, and number of weeks; (5) research outcomes. The PEDro scale was utilised to assess the quality of included articles. This reliable methodological quality assessment tool [26] comprises 11 items designed to evaluate aspects such as randomisation, blinding, group comparability, and statistical analysis [27]. Each item is scored as either “yes” (1) or “no” (0). Scores range from 0 to 10, with higher scores typically indicating higher quality [28]: 6–10 points suggest high quality, 4–5 points indicate moderate quality, and ≤ 3 points indicate poor quality. The assessment using the PEDro scale was independently conducted by two researchers. In cases of disagreement, a third researcher was consulted to reach a consensus [29]. Please refer to Table 2.

**Table 2:** Summary of methodological quality assessment scores

References	Eligibility criteria	Random allocation	Concealed allocation	Group similar at baseline	Blind subject	Blind therapist	Blind assessor	Follow-up	Intention-to-treat analysis	Between-group comparisons	Point measure and variability	PEDro score
Birimoglu Okuyan and Deveci [30]	1	1	1	1	0	0	0	1	1	1	1	7
Yan, Yang [31]	1	1	0	1	0	0	0	0	1	1	1	5
Chewning, Hallisy [32]	1	1	0	1	0	0	0	1	1	1	1	6
Day, Hill [33]	1	1	0	1	0	0	1	0	1	1	1	6
Ni, Mooney [34]	1	1	0	1	0	0	0	0	1	1	1	5
Lifeng, Jun [35]	1	1	0	1	0	0	0	0	1	1	1	5
Zhang, Ishikawa-Takata [36]	1	1	0	1	0	0	0	0	1	1	1	5
Li, Harmer [37]	1	1	0	1	1	0	1	1	1	1	1	8
Liang [38]	1	1	0	1	0	0	0	0	1	1	1	5
Taylor, Hale [39]	1	1	0	1	0	0	0	1	1	1	1	6
Gao, Leung [40]	1	1	0	1	1	0	0	1	1	1	1	7
Liao, Liu [41]	1	1	0	1	1	1	1	0	1	1	1	8
Zhu, Peng [42]	1	1	0	1	0	0	0	0	1	1	1	5
Hwang, Chen [43]	1	1	1	1	0	0	0	1	1	1	1	7
**Total**	14	14	2	14	3	1	3	6	14	14	14	

## Results

### Study selection and characteristics

**Fig. 1:**
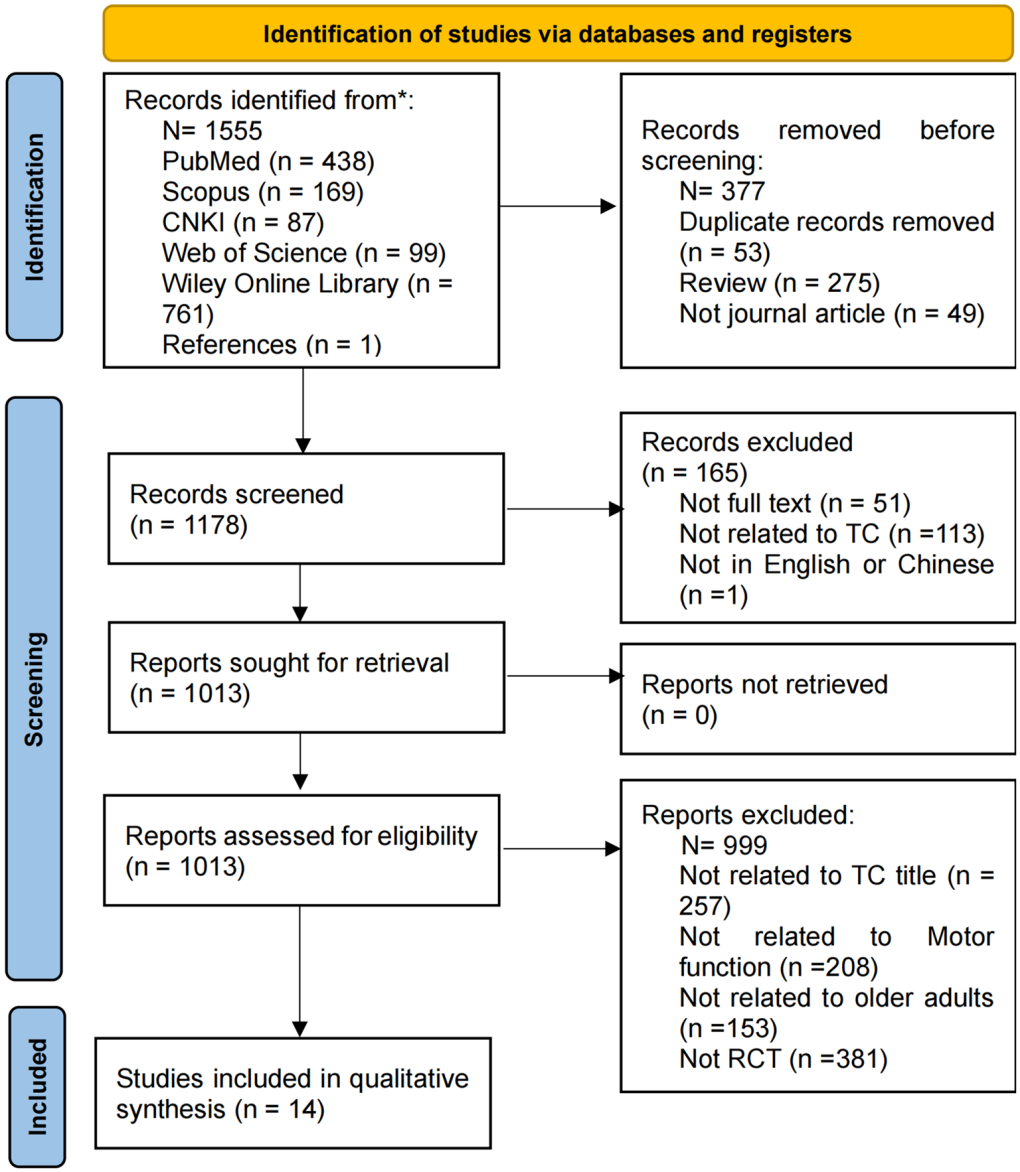
Prisma study selection process

Refer to Fig. 1 for the study selection process. A total of 1555 articles were initially retrieved from electronic databases: PubMed (438), Scopus (169), CNKI (87), Web of Science (99), Wiley Online Library (761), and references (1). To streamline the selection process, we used literature management software to automatically remove duplicate entries. Two reviewers (Fan and Kim) then independently screened the articles to retain only those relevant and of high quality for inclusion in this review. Following this, 257 articles were excluded based on title alone, while articles unrelated to motor function (208 articles), not focused on older adults (153 articles), and those not classified as RCTs (381 articles) were further excluded. This process resulted in a final selection of 14 articles for detailed analysis [30–39, 40–43].

### Study quality assessment

Two researchers independently assessed the 14 articles using the PEDro scale. The assessment results were as follows: Random allocation (*n* = 14), Group similar at baseline (*n* = 14), Between-group comparisons (*n* = 14), Intention-to-treat analysis (*n* = 14), Point measure and variability (*n* = 14), Blind assessor (*n* = 3), Blind subject (*n* = 3), Blind therapist (*n* = 1), Follow-up (*n* = 6), and Concealed allocation (*n* = 2). According to the PEDro scale, the quality of the articles included in this study was rated as high (*n* = 8) or moderate (*n* = 6), with scores ranging from 5 to 8. This indicates a generally high methodological quality among the selected articles, supporting the reliability of this review findings. For detailed results and further information on quality assessment, please refer to Table 2.

### Characteristics of included studies

The characteristics of the population included in this study are shown in Table 3. A total of 14 studies were included, focusing on the motor function of older adults. The specific characteristics are as follows:

- **Total sample size**: 3009 participants.
- **Gender**: 12 studies included both males and females [30, 32–38, 40, 41, 43, 39]; one study focused on women [31]; one study described only males [42].
- **Age**: All participants were over 60 years old. 12 studies included participants aged 60–75 years [30–36, 38, 40, 41, 43, 39]; two studies included participants over 75 years [37, 42].
- Description of participants health conditions: Two studies involved MCI populations [30, 41]; five studies addressed the risk of falls [32, 34, 37, 43, 39]; and other health conditions were represented as follows: NS-LBP [31], disability [33], chronic disease [35], poor balance [36], OA [38], PD [40], and sarcopenic [42].

**Table 3:** Sample features, main outcomes, and participants’ characteristics

References	Participants (*N*/G/A)	Sample features	Intervention	TC Style	TC Form	Comparison	Outcomes
Birimoglu Okuyan and Deveci [30]	*N* = 20, G = M/F Age = 74.21 ± 6.93	MCI	EG: TC CG: Untrained	Yang style	Not mentioned	EG: 12wk/2/35–40 min CG: not applicable	Balance↑ (TAT), Gait ↑ (TAT), Falls behavioral ↓ (FaB)
Taylor, Hale [39]	*N* = 684, G = M/F Age ≈ 74.5	Risk of falling	EG1: TC EG2: TC CG: LLE	Sun style	10-form	EG1: 20wk/1/1hour EG2: 20wk/2/1hour CG: 20wk/1/1hour+	Balance↑ (ST), strength ↑ (CST), Mobility↔ (TUGT)
Yan, Yang [31]	*N* = 20, G = F Age > 65	NS-LBP	EG: TC CG: No exercise	Chen style	18-form	EG: 6wk/3/1 hour CG: No exercise	Balance↑ (dynamic), Gait↑ (stride width, gait velocity, stride length)
Chewning, Hallisy [32]	*N* = 242, G = M/F Age ≥ 65	History of falling	EG: TC CG: not mentioned	Yang style	Not mentioned	EG: EG:6-wk/2/90 min CG: no exercise intervention	Balance↑ (SL), Gait↑ (TUGT), Mobility↑ (TUGT), Strength↑ (CST)
Day, Hill [33]	*N* = 503, G = M/F Age = 70.8 ± 6.3	Preclinically disabled	EG: TC CG: flexibility and stretching	Sun style	46-form	EG: 24wk/2/60min CG: 24wk/2/60min	Balance ↔ (SL), Mobility ↔ (TUGT), Strength ↔ (ST), Gait ↔ (DW)
Ni, Mooney [34]	*N* = 39, G = M/F Age = 74.15 ± 6.99	History of falling	EG1: TC EG2: Balance training CG: Yoga	Chen style	18-form	EG: 12wk/2/60min EG: 12wk/2/60min CG: 12wk/2/60min	Balance↑ (SL), Gait ↑ (8UG, Walking speed)
Lifeng, Jun [35]	*N* = 60, G = M/F Age = 65.35 ± 7.76	Chronic disease	EG: TC CG: Lower limb bike	Not mentioned	42-form	EG:12wk/5/30–35 min CG: 12wk/5/30–35 min	Gait↑ (6MWT), Balance↑ (SPPB), Strength↑ (CST)
Zhang, Ishikawa-Takata [36]	*N* = 49, G = M/F Age ≥ 60	Poor balance	EG: TC CG: No intervention	Yang style	24-form	EG: 8wk/7/60min CG: No intervention	Balance↑(SL), Gait↑ (10 MW), FOF↓ (FES score)
Li, Harmer [37]	*N* = 670, G = M/F Age = 77.5 ± 5.6	History of falling	EG1: TC EG2: Multimodal exercise CG: Stretching exercise	Not mentioned	8-form	EG1: 24wk/2/60min+ EG2: 24wk/2/60min CG: 24wk/2/60min	Fall rate↓
Liang [38]	*N* = 91, G = M/F Age = 60–70	OA	EG1: TC EG2: Resistance training CG: No intervention	Yang style	24-form	EG1: 16wk/4/60min+ EG2: 16wk/4/60min CG: 24wk/4/60min	Gait↑(6WMT), Mobility↑ (TUGT)
Gao, Leung [40]	*N* = 76, G = M/F Age = 69.54 ± 7.32	PD	EG: TC CG: No intervention	Yang style	24-form	EG: 12wk/3/60min CG: 12wk/3/60min	Balance ↑ (BBS), Mobility↑ (TUGT), Fall rate↓
Liao, Liu [41]	*N* = 20, G = M/F Age ≥ 65	MCI	EG: tDCS + TC CG: sham + TC	Yang style	24-form	EG: 12wk/3/40min CG: 12wk/3/40min	Gait performance↑
Zhu, Peng [42]	*N* = 79, G = M Age ≥ 85	Sarcopenic	EG1: TC + Health Education EG2: WBV + Health Education CG: Health education	Simplify	8-form	EG1: 8wk/5/40min EG2: 8wk/5/40min CG: 8wk/1/month/40min	Balance↑(SL), Gait↑ (6WMT), mobility↑ (TUGT), strength↑ (FTSST, handgrip),
Hwang, Chen [43]	*N* = 456, G = M/F Age = ≥ 60	History of falling	EG: TC CG: LET	Yang style	18-form	EG: 24wk/1/60min CG: 24wk/1/60min	Balance↑ (TAT), Strength↑ (Handgrip), Fall rate↑ (FES)

Note N: number of samples; G: gender; A: age; M: male; F: female; EG: experimental group; CG: control group; WK: week, ↓significantly decreased; significantly increased ↑; no significant change ↔; MCI: mild cognitive impairment; LLE: low-level exercise; FES: falls efficacy scale; OA: chronic symptomatic hip or knee osteoarthritis PD: parkinson’s disease; tDCS: transcranial direct current stimulation; WBV: whole-body vibration; M: month; FOF: fear of fall; NS-LBP: non-specific low back pain; TAT: Tinetti scale; FaB: falls behavioral scale; ST: step test; CST: chair stand test; TUGT: time up and go test; SL: single leg balance; DW: Distance walked; 8UG: 8-foot up-and-go test; 6MWT: 6 min walk test; SPPB: short physical performance battery; 10WM: 10 m walking; FES: Falls Efficacy Scale; FTSST: five-times-sit-to-stand test

### Intervention characteristics

Intervention characteristics included intervention frequency, duration, and period. All 14 studies employed TC interventions, with two studies incorporating combined interventions [41, 42]. Nine studies compared two groups [30–33, 35, 36, 40, 41, 43], while five studies conducted three-group comparisons, including two experimental groups and one control group [34, 37, 38, 42, 39].

The intervention cycles varied as follows:

- Five studies utilised 12-week interventions [30, 34, 35, 40, 41].
- Three studies had 24-week intervention periods [33, 37, 43].
- Two studies implemented eight-week interventions [36, 42].
- Two studies conducted six-week interventions [31, 32].
- One study had a 16-week intervention [38].
- One study implemented a 20-week intervention [39].

### Outcomes

This section includes the research results of 14 articles, all of which used TC as the intervention method and reported the effects on motor function in older adults with functional impairments.

### Effects of different Tai Chi interventions on balance

eleven studies examined balance outcomes [30–36, 39, 40, 42–43]. Regarding TC intervention styles used for balance, there was variation: five studies used Yang-style TC [30, 32, 36, 40, 43], two studies used Sun-style TC [33, 39], two studies used Chen TC [31, 34], one study used simplified TC [42], and one studies did not specify the TC style [35]. Notably, one study utilising Sun-style TC did not show improvement in balance outcomes [33].

Regarding the populations studied for balance:

- Five studies assessed balance in individuals at risk of falls [32, 34, 37, 43, 39].
- There was one study assessing balance in individuals with MCI, NS-LBP, chronic disease, poor balance, PD, and sarcopenia.
- One study assessed balance in individuals with preclinically disabled, showing no significant improvement [33].

The following methods were used to assess balance: six studies employed the single-leg balance stand test (SL), a common measure of balance. Most studies reported improved SL test scores following TC intervention, with only one study showing no improvement. Additionally, two studies used the Tinetti Assessment Tool (TAT) scale, both of which indicated enhanced balance. Two other articles applied the Short Physical Performance Battery (SPPB) and the Berg Balance Scale (BBS), each showing improvement in balance scores. In another study focusing on dynamic balance, the specific test method was not specified. However, most studies did not clearly distinguish whether these assessment methods measured dynamic or static balance.

### Effects of different Tai Chi interventions on gait

Gait function was evaluated in ten studies [30–36, 38, 41, 42]. The interventions included various styles of TC: five studies used the Yang style [30, 32, 36, 38, 41], one study used the Sun style [33], two studies used the Chen style [31, 34], one study used the Simplified style [42], and one study did not specify the TC style [35].

Specifically, gait function was assessed in:

- Two studies involving individuals with MCI [30, 41].
- Two studies involving individuals at risk of falls [32, 34].
- One study evaluated gait in patients with chronic disease [35].
- One study assessed gait function in individuals with Preclinically disabled, showing no significant improvement [33].

Gait outcomes were measured using the following methods: the 6-minute walk test (6MWT), 10-metre walk test (10MWT), 8-foot up-and-go test (8UG), Timed Up and Go test (TUGT), TAT, and Distance Walked (DW). The 6MWT, used in three studies, was the most common method for assessing gait [35, 38, 42], as it typically evaluates gait speed and provides insight into an individual walking stability [44]. The remaining tests were each used in only one study, with most indicating improvement. However, only one DW test did not show a positive effect on gait ability [33]. Additionally, only one study comprehensively reported gait indicators, including stride width, gait velocity, and stride length [31].

### Effects of different Tai Chi interventions on mobility

Six studies reported on mobility [32, 33, 38, 40, 42, 39]. The interventions included two studies using Sun-style TC [33, 39], three studies using Yang-style TC [32, 38, 40], and one study using Simplified-style TC [42].

Mobility function was mainly reported in:

- Two studies involving individuals at risk of falls [32, 39].
- One study involving individuals with preclinically disabled, showing no significant improvement [33].
- There was one study each involving individuals with OA, PD, and sarcopenia.

This study did not find reports on mobility for individuals with MCI, NS-LBP, chronic disease, or poor balance. It is worth noting that TUGT is a widely used tool to measure activity.

### Effects of different Tai Chi interventions on strength

Six studies reported on strength function. These included two studies on Sun style TC [33, 39], two studies on Yang style TC [32, 43], one study without specifying the TC style [35], and one study on Simplified style TC [42]. Additionally, this review identified three studies involving individuals at risk of falls [32, 43, 39], one study each on chronic disease and sarcopenia, and one study on individuals with disabilities, though the results were not positive [33].

Four studies focused exclusively on measuring lower body muscle strength in older adults. Of these, three used the Chair Stand Test (CST) [32, 35, 39], and one applied the Step Test (ST) [33]. Two studies examined upper limb strength [42, 43]. Most studies focused on either upper or lower limb strength, with only one study conducting a comprehensive assessment of both [42]. Notably, grip strength was the primary measure for upper limb strength, while the CST was the commonly used method for assessing lower limb strength.

### Effects of different Tai Chi interventions on falls

Five studies are related to falls [30, 36, 37, 40, 43]. Among them, four studies focused on Yang style TC [30, 32, 36, 43], and two studies did not specify the TC style [37]. The populations studied include individuals with MCI [30], individuals with poor balance [36], individuals with a history of falls [37], and individuals with PD [40].

The Falls Efficacy Scale (FES) is a commonly used tool for measuring fall risk [36, 43], and two studies included in this review employed this measurement. Another study utilised the Falls Behavior Scale (FaB) to assess falls in a population with mild cognitive impairment (MCI) [30]. Additionally, two studies reported instances of falls without specifying the methods used [37, 40].

## Discussion

This study aimed to examine the effects of various TC interventions on motor function in older adults with functional impairments. Our comparison of different intervention groups revealed that the distinct TC styles had varied effects on motor function, including balance, gait, mobility, strength, and fall prevention. Unlike other reviews, this study not only evaluated the overall benefits of TC but also compared the specific effects of different styles (Yang, Sun, Chen, and simplified forms) across diverse populations.

Fourteen articles employed various styles of TC interventions, including Yang, Sun, Chen, and simplified styles. These interventions encompassed six distinct TC movement forms: 8-form, 10-form, 18-form, 24-form, 42-form, and 46-form. TC has demonstrated positive effects on many outcome measures of motor function, particularly in improving balance, gait, mobility, and strength in older adults [45]. However, differences exist among the various TC styles.

Yang style TC was the most frequently used, appearing in a total of seven studies [30, 32, 36, 38, 40, 41, 43]. Specifically, four studies focused on the 24-movement form of Yang style TC [36, 38, 40, 41], while one study examined the 18-movement form Hwang, Chen [43]. Additionally, two studies featured the Chen style with its 18 movements Yan, Yang [31, 34], while the Sun style was represented by two studies using the 10-movement and 46-movement forms [34, 39], respectively. The simplified style included one study 8-movement forms [42]. Four studies did not specify the TC style or form used. Consequently, this review suggests that Yang-style 24-movement TC positively affects the motor function of individuals with PD, MCI, OA, and poor balance. This finding aligns with previous research indicating that Yang-style 24-movement TC can enhance physical function and significantly reduce fall rates in older adults who have experienced a stroke [46].

In this study, we investigated the effects of different styles of TC on motor function in older adults with functional impairments. Variations among TC styles can lead to differences in movement patterns, resulting in diverse exercise outcomes [47]. Four primary TC styles were included in this research. Yang-style TC, characterised by slow and smooth movements, has been shown to enhance balance and coordination, which aligns with our findings [48]. Specifically, studies indicate that Yang-style TC can improve balance in individuals with a history of falls, PD, OA, poor balance, and MCI.

Sun-style TC is recognised for its light movements, which demand agility and flexibility from practitioners [49]. Our results demonstrated that Sun-style TC can enhance balance and strength in older adults with a history of falls; however, another study involving individuals with preclinical disabilities did not report significant improvements in motor function. This discrepancy may arise from the complex movements inherent in Sun-style TC, including jumping and spinning, which require a higher level of balance and strength [50].

Chen-style TC, the oldest form, features more intricate and intense movements compared to Yang and Sun styles [51, 52]. It emphasises low postures, body coordination, and flexibility. Our study found that Chen-style TC can improve balance and gait measures in individuals with a history of falls and NS-LBP. Additionally, we examined simplified TC, which has been adapted to accommodate practitioners’ physical conditions. The results indicated that simplified TC can also enhance balance, flexibility, and strength in individuals with sarcopenia.

The characteristics of the participants in the included studies played a pivotal role in influencing the outcomes. This review focused on older adults with different levels of functional impairment, including PD, OA, MCI, preclinical disability, chronic illness, sarcopenia, and fall-related older adults. They may respond differently to TC interventions based on their baseline functional capacity, severity of impairment, and overall health. For instance, studies involving individuals with milder functional impairment generally reported greater improvements in balance and gait. In contrast, one study of Sun-style 46-posture TC in preclinically disabled people did not observe improvements in outcome measures. In general, the quality and intensity of TC practice also affect the results; higher quality, more intensive programs may lead to better results.

In terms of balance function, Yang style TC, especially the 24-posture TC, was the most effective. This finding is consistent with the results of [53], who also pointed out that Yang style TC significantly improved the balance ability of older adults. Balance is influenced by several key factors, including strength, proprioception, flexibility, and coordination [54]. Yang-style 24-posture Tai Chi incorporates slow movements and encourages a low center of gravity in the lower limbs during practice [55]. These elements contribute to enhancing the practitioner’s muscle strength and stability when standing on one leg. Given these considerations, the results of this study support the use of Yang-style 24-posture Tai Chi as the primary intervention for improving balance. In addition, a 12-week, 2–7 sessions, 35–60 min each time intervention is recommended.

In terms of gait improvement, Yang-style TC consistently outperformed other styles, aligning with findings by [56], who reported similar benefits. Meanwhile, both Chen-style and simplified TC demonstrated significant improvements in gait. Chen-style TC combines slow and fast movements to enhance muscle strength, power, and endurance [57]. It includes explosive movements, known as “fa jin,” which engage fast-twitch muscle fibres, promoting better muscle activation and coordination [58]. This dynamic characteristic of Chen-style TC aids practitioners in better controlling their gait mechanics. In contrast, Sun-style TC did not yield consistent improvements in gait, underscoring the importance of selecting a specific TC style to effectively address gait issues. This review still recommends an intervention duration of 12 weeks, with a frequency of 2–3 sessions per week, each lasting 35–60 min.

Our review also identified Yang-style TC as the most effective for enhancing mobility, while a simplified eight-movement form of TC also demonstrated positive effects. The Timed Up and Go Test (TUGT) is the most commonly used method to assess mobility; it involves a series of tasks that measure gait performance, balance, and overall mobility [59]. This review found that Yang-style TC can significantly improve mobility in individuals with a history of falls, OA, and PD. Furthermore, the simplified eight-movement TC proved beneficial for mobility in the sarcopenic population. Based on these findings, we recommend that older adults with a history of falls engage in Yang-style 24-movement TC to enhance their mobility. In contrast, Sun-style TC did not yield significant improvements in mobility.

Strength can serve as a key indicator of health status. Generally, reduced strength is linked to various negative outcomes, such as falls, disability, and even mortality [60, 61]. The findings from this study indicate that both Yang-style and simplified TC positively impact strength indicators, while Sun-style Tai Chi has beneficial effects specifically for older adults with a history of falls. However, it appears to have no effect on those who are preclinically disabled.

Practising TC engages all the muscles of the body, and as training intensity increases, muscle activation is enhanced throughout [62]. It is important to note that the Chen-style TC intervention did not include strength indicators, indicating that further research is needed to confirm its effects on strength in older adults with functional disabilities. Based on the results of this study, we continue to recommend Yang-style TC as an effective means of improving strength indicators, particularly for older adults with a history of falls.

Fall-related issues are a prevalent concern among older adults. Four studies have shown that Yang-style TC can effectively reduce the incidence of falls in individuals with various functional disabilities, including MCI, poor balance, PD, and a history of falls. Practising TC involves focused, deliberate movements that enhance proprioception [63]. Improved proprioception allows individuals to quickly adjust their posture and respond effectively to sudden changes, which are essential skills for preventing falls [64]. To effectively reduce fall rates, this study recommends an intervention duration of eight to 24 weeks, with sessions lasting 35 to 60 min each.

### Study limitations

Several limitations should be acknowledged in this review.

First, while this study included various TC intervention methods, some styles were underrepresented. Greater attention should be given to these less frequently studied TC types in future research.

Additionally, the sample sizes for certain populations with functional impairments were small. For instance, studies involving individuals with nonspecific low back pain, preclinical disabilities, or chronic diseases were limited. Future research should aim to encompass these groups more comprehensively to enhance the generalizability of the findings.

## Conclusion

This systematic review investigates the effects of Yang-style, Chen-style, Sun-style, and simplified-style TC on motor function in older adults with functional impairments. The results demonstrate that Yang-style TC, particularly the 24-movement form, significantly enhances balance, gait, mobility, and strength in individuals with MCI, a history of falls, poor balance, OA, and PD. Additionally, Chen-style TC (18 movements) is effective in improving balance and gait for those with NS-LBP and a history of falls. In contrast, Sun-style TC did not produce significant improvements in motor function among individuals with preclinical disabilities. Moreover, there is a notable scarcity of research on simplified-style TC, indicating a need for further exploration in this area. The review utilised various assessment tools, including the SL for balance, the 6MWT for gait, TUGT for mobility, CST for strength, and the FES. Finally, this study suggests that a TC intervention duration of 12 weeks, performed two to five times per week, is recommended to achieve optimal outcomes.

## Electronic supplementary material

Below is the link to the electronic supplementary material.

Supplementary Material 1

## Data Availability

No datasets were generated or analysed during the current study.
