# Supplementary material for: Effects of different types of Tai Chi intervention on motor function in older adults: a systematic review
Source: Aging Clin Exp Res. 2025 Jan 22;37(1):32. doi: 10.1007/s40520-024-02894-5 (PMC11754367; doi:10.1007/s40520-024-02894-5)
Supplement: Supplementary file 1 — Supplementary Material 1 [file 40520_2024_2894_MOESM1_ESM.docx]

| Keywords | The keywords were: “Tai Ji” OR “Tai-ji” OR “Tai Chi” OR “Chi Tai” OR “Tai Ji Quan” OR “Ji Quan Tai” OR “Quan Tai Ji” OR “Taiji” OR “Taijiquan” OR “T'ai Chi” OR “Tai Chi Chuan” AND “motor” “movement” OR “motion” OR “mobility” OR “function” OR “performance” AND “old people” OR “elderly” OR “senior*” OR “old adult*” OR “aged” OR “older people” OR “older adults” OR “geriatric” | |
| --- | --- | --- |
| **Database** | **Strategy** | **Results** |
| PubMed  （From inception to 5.09.2024） | **Search: (("Tai Ji"[Title/Abstract] OR "Tai-ji"[Title/Abstract] OR "Tai Chi"[Title/Abstract] OR "Chi Tai"[Title/Abstract] OR "Tai Ji Quan"[Title/Abstract] OR "Ji Quan Tai"[Title/Abstract] OR "Quan Tai Ji"[Title/Abstract] OR "Taiji"[Title/Abstract] OR "Taijiquan"[Title/Abstract] OR "T'ai Chi"[Title/Abstract] OR "Tai Chi Chuan"[Title/Abstract]) AND ("motor" "movement"[Title/Abstract] OR "motion"[Title/Abstract] OR "mobility"[Title/Abstract] OR "function"[Title/Abstract] OR "performance"[Title/Abstract])) AND ("old people"[Title/Abstract] OR "elderly"[Title/Abstract] OR "senior*"[Title/Abstract] OR "old adult*"[Title/Abstract] OR "aged"[Title/Abstract] OR "older people"[Title/Abstract] OR "older adults"[Title/Abstract] OR "geriatric"[Title/Abstract])** | 438 |
| SCOPUS (From inception to 05.09.2024) | ( "Tai Ji" OR "Tai-ji" OR "Tai Chi" OR "Chi Tai" OR "Tai Ji Quan" OR "Ji Quan Tai" OR "Quan Tai Ji" OR "Taiji" OR "Taijiquan" OR "T'ai Chi" OR "Tai Chi Chuan" AND "motor" "movement" OR "motion" OR "mobility" OR "function" OR "performance" AND "old people" OR "elderly" OR "senior*" OR "old adult*" OR "aged" OR "older people" OR "older adults" OR "geriatric" ) | 169 |
| CNKI (From inception to 22.06.2024) | [(标题、关键词和摘要=太极 + 太极拳) 和 (标题、关键词和摘要=运动功能 + 运动能力) 和 (标题、关键词和摘要=老年人 + 老人)](https://www.cnki.net/kns/AdvSearch?id=30&dbcode=CFLS&searchtype=gradeSearch&ishistory=1) | 87 |
| Web of Science (From inception to 5.09.2024) | https://www.webofscience.com/wos/alldb/summary/fbfb797e-97f0-40ab-a3a9-2dac2cbf650d-xxxxxxxx/relevance/1#1 AND #2 AND #3 and Preprint Citation Index (Exclude – Database)  “Tai Ji” OR “Tai-ji” OR “Tai Chi” OR “Chi Tai” OR “Tai Ji Quan” OR “Ji Quan Tai” OR “Quan Tai Ji” OR “taijin” OR “taichiquan” OR “T'ai Chi” OR “Tai Chi Chuan” (Title) AND “motor” “movement” OR “motion” OR “mobility” OR “function” OR “performance” (Title) AND “old people” OR “elderly” OR “senior*” OR “old adult*” OR “aged” OR “older people” OR “older adults” OR “geriatric” (Title) and Preprint Citation Index (Exclude – Database)tabase) | 99 |
| Wiley Online Library  (From inception to 5.09.2024) | **763**results for**"“Tai Ji” OR “Tai-ji” OR “Tai Chi” OR “Chi Tai” OR “Tai Ji Quan” OR “Ji Quan Tai” OR “Quan Tai Ji” OR “Taiji” OR “Taijiquan” OR “T'ai Chi” OR “Tai Chi Chuan”"** anywhere and **"“motor” “movement” OR “motion” OR “mobility” OR “function” OR “performance”"** anywhere and **"“old people” OR “elderly” OR “senior*” OR “old adult*” OR “aged” OR “older people” OR “older adults” OR “geriatric”"** anywhere | 761 |
| Total |  |  |
| Final Results | References 1 | 14 |
